# Human Texture Vision as Multi-Order Spectral Analysis

**DOI:** 10.3389/fncom.2021.692334

**Published:** 2021-07-26

**Authors:** Kosuke Okada, Isamu Motoyoshi

**Affiliations:** Department of Life Sciences, The University of Tokyo, Tokyo, Japan

**Keywords:** vision, texture, image statistics, frequency analysis, psychophysics, human

## Abstract

Texture information plays a critical role in the rapid perception of scenes, objects, and materials. Here, we propose a novel model in which visual texture perception is essentially determined by the 1st-order (2D-luminance) and 2nd-order (4D-energy) spectra. This model is an extension of the dimensionality of the Filter-Rectify-Filter (FRF) model, and it also corresponds to the frequency representation of the Portilla-Simoncelli (PS) statistics. We show that preserving two spectra and randomizing phases of a natural texture image result in a perceptually similar texture, strongly supporting the model. Based on only two single spectral spaces, this model provides a simpler framework to describe and predict texture representations in the primate visual system. The idea of multi-order spectral analysis is consistent with the hierarchical processing principle of the visual cortex, which is approximated by a multi-layer convolutional network.

## Introduction

The primate visual system rapidly analyzes texture information, or image statistics or ensemble, from complex natural images (Landy and Graham, 2004; Rosenholtz, 2014; Whitney and Yamanashi Leib, 2018), and uses it for the immediate perception and recognition of scenes, objects, and surface materials (Lowe, 1999; Oliva and Torralba, 2001, 2006; Motoyoshi et al., 2007; Rosenholtz et al., 2012). Recent studies further suggest that our perception in the peripheral vision is generally governed by such texture information (Balas et al., 2009; Freeman and Simoncelli, 2011). Neural and computational models of texture processing are thus important for understanding the nature of visual cognition.

Visual texture is defined as the image region consisting of complex repetition of various features (Bergen, 1991). By this definition, visual perception of a texture is determined by the global distribution of features, without positional information about the features within the region. The pioneering works by Julesz (1965) and later psychophysical studies (Regan, 2000; Landy and Graham, 2004) suggest that the human visual system encodes such global measures only for low-level features in most cases, although there are some cases in which textures can be discriminated on the basis of higher-level features (Julesz, 1981; Motoyoshi and Kingdom, 2010).

Following Julesz’s conjecture, studies have proposed a computational model that analyzes spatial distribution of low-level statistics. The most influential one is often referred to as the Filter-Rectify-Filter (FRF) model (Bergen and Adelson, 1988; Bergen and Landy, 1991). The FRF model consists of two stages of image processing based on spatial filtering and energy computation. At the 1st stage, bandpass filters decompose the luminance image into different orientation and spatial frequency subbands, and the non-linear computation converts them to energy representation. The 2nd stage repeats the same computation for each subband energy images. The final output is assumed to be a spatial summation of the 2nd-order energies. A large number of psychophysical evidence shows that this simple model (Bergen and Adelson, 1988; Landy and Graham, 2004), and its modified versions (Malik and Perona, 1990; Motoyoshi and Kingdom, 2007), can explain human performance on texture discrimination tasks. On the other hand, there is another representative model of texture vision called the Portilla-Simoncelli (PS) statistics model (Portilla and Simoncelli, 2000), which is becoming prevalent in visual neuroscience (Freeman and Simoncelli, 2011; Freeman et al., 2013; Okazawa et al., 2015). The PS statistics model computes the statistical properties of subband responses and their relationship, and can predict the perception of natural textures based on the ensemble (Portilla and Simoncelli, 2000; Balas et al., 2009; Freeman and Simoncelli, 2011; Rosenholtz et al., 2012).

Revisiting the computational architecture of the FRF model, the present study proposes a novel model, or a viewpoint, that natural texture perception is essentially based on 1st- and 2nd-order spectral analyses. We show that the computations of this model are functionally consistent with the computations of PS statistics in two single-frequency spaces. To validate the model, we also introduce a novel texture synthesis based only on scrambling of the 1st- and 2nd-order phase spectra.

## Texture Processing as Two-Stage Spectral Analysis

**FIGURE 1:**
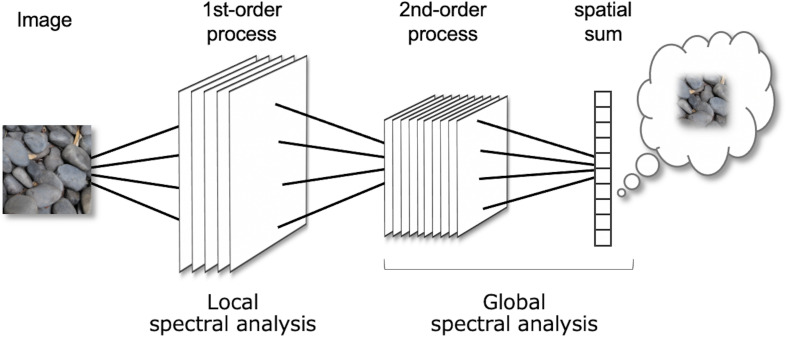
A diagram of the Filter-Rectify-Filter (FRF) model of texture vision. The model can be regarded as a two-stage amplitude spectral analysis: The 1st stage is a local spectral analysis of the luminance input, and the 2nd stage is a global spectral analysis of the 1st-stage output.

Figure 1 shows a very simplified architecture of the FRF model. As described above, each stage consists of spatial filtering and energy computation. Conceptually, these operations correspond to a Fourier analysis and the local observation of the amplitude. In this view, the 1st-order process is regarded as a local spectral analysis of the luminance image, and the 2nd-order process is a spectral analysis of the 1st-order energy outputs for each subband. The model assumes that perception is determined by a global measure (e.g., the spatial sum) from the localized 2nd-order process over space. The spatial sum is computed by pooling signals within a receptive field large enough to cover the entire texture region (In the conventional FRF model, this pooling is often assumed in the decision process). Therefore, a set of the 2nd-order process and following spatial pooling can be approximated as a global, not local, spectral analysis. Note that “global spectrum” does not mean spectrum of the entire visual field, just as “global image statistics” in the PS model do not mean image statistics of the entire visual field.

**FIGURE 2:**
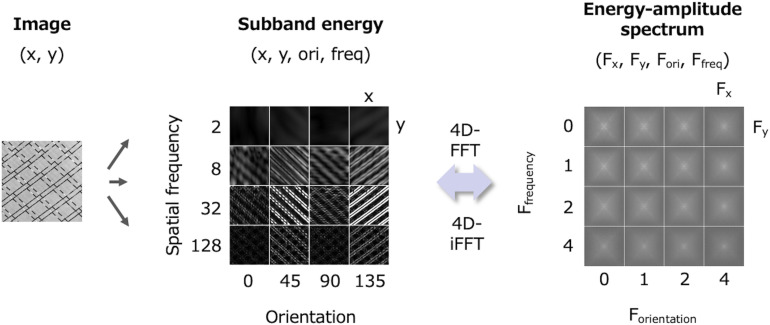
Relationship between subband energy data in the space domain (x, y, ori, and freq) and its amplitude spectrum in the frequency domain (Fx, Fy, Fori, and Ffreq).

The conventional FRF model assumes that both the 1st- and 2nd-order processes involve two-dimensional filtering only for space (x,y). However, the energy output of the 1st-order process is four-dimensional, consisting of space (x,y), orientation (ori), and spatial frequency (freq). Corresponding to the dimensionality of the output, the 2nd-order process must be a spectral analysis of four dimensions (x, y, ori, and freq). Figure 2 illustrates a four-dimensional subband energy in the space domain (x, y, ori, and freq) and its amplitude spectrum in the Fourier domain (Fx, Fy, Fori, and Ffreq) (Motoyoshi and Kingdom, 2003).

From a functional view, this notion is consistent with another powerful texture model, the PS statistics model (Portilla and Simoncelli, 2000). The PS statistics model involves subband decomposition and energy measurement similar to the FRF model, and a variety of image statistics are measured at each stage. An ensemble of these PS statistics then determines the texture perception. It is well known that by matching the PS statistics of a noise to those of a target texture, one can synthesize a perceptually similar texture, strongly supporting the validity of PS statistics in natural texture perception. However, the PS statistics model is relatively complex as it considers many different classes of statistics, ranging from low-level statistics such as histogram moments and the power of subbands, to high-level ones such as autocorrelation/cross-correlation of the linear and energy subbands. For the cross-correlation of the energy subbands, both cross-orientation and cross-scale are considered. However, by viewing the energy as four-dimensional data (Figure 2, left), these cross-correlations are considered as an autocorrelation along the orientation and spatial frequency dimensions. Thus, multiple classes of energy-related PS statistics can be summarized into one class as a four-dimensional (x, y, ori, and freq) autocorrelation. Given that the Fourier transform of an autocorrelation function results in a power spectrum, the energy autocorrelation is represented as the 4D amplitude spectrum (Figure 2, right). This means that the energy spectrum functionally corresponds to the higher-order PS statistics. In the same way, the autocorrelation of linear subbands corresponds to the luminance spectrum. The two-stage spectrum is closely related to the PS statistics model as well as to the extended FRF model, and it enables us to deal with the two prevailing texture models in the frequency domain.

**FIGURE 3:**
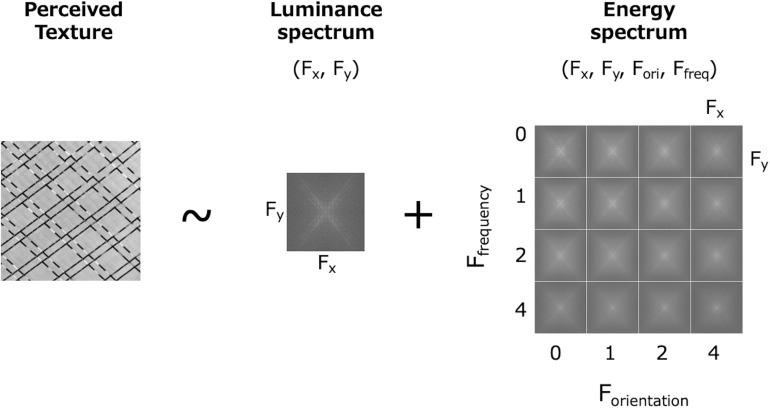
A model in which texture perception is based on the 1st- and 2nd-order frequency spectrum. The 1st-order is the spectrum of the luminance image (2D) and the 2nd-order is the spectrum of the subband energies (4D).

In summary, the FRF model can be extended and considered as a simple Fourier spectral analysis of the luminance data (1st-order, 2D) and the subband energy data (2nd-order, 4D). On this basis, we propose a novel model that states visual texture processing is represented as 1st- and 2nd-order spectral analyses (Figure 3). From this viewpoint, the 1st-order spectrum has a detailed frequency representation of the luminance image, including a wide range of periodic variations, and the 2nd-order spectrum has a 4D frequency representation of the subband energy data. The phase information (e.g., the total power in each subband) is lost in the 2nd-order spectrum but implicitly represented in the 1st-order spectrum.

## Luminance-Energy Phase Randomized Image

Synthesis of a natural texture based on a model is a powerful and ecologically valid way to test the model. One of the most successful cases is the PS synthesis (Portilla and Simoncelli, 2000; Balas, 2006). To test the two-stage spectrum model, we attempted to generate synthetic natural textures based on only two spectra. Actually, we simply randomized the phase of the original image while preserving the original luminance and energy amplitude spectra. Here we call this the luminance-energy phase randomization (lum-energy PR) (The present model does not consider the perception of artificial textures composed of dots and lines because they are ecologically invalid).

**FIGURE 4:**
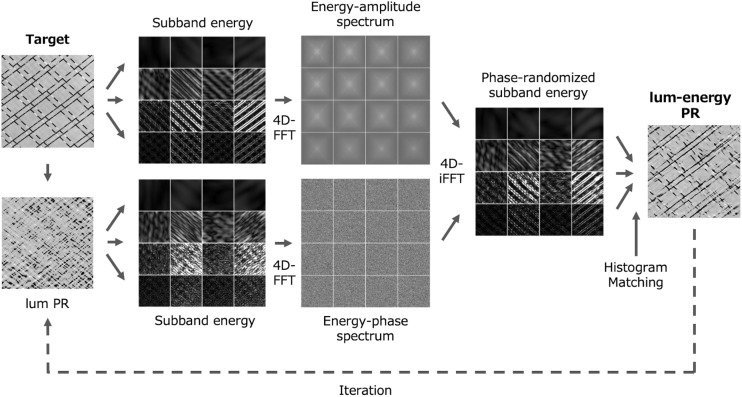
Schematic diagram of the luminance-energy phase randomization. For simplicity, only four orientations and four scales are shown.

The luminance-energy phase randomized image is generated as shown in Figure 4. Since the data is represented in only two spaces (1st- and 2nd-order spectra), the processing is very simple. Each step proceeds as follows (see section “Methods: Luminance-Energy Phase Randomization” in more detail).

(1) Using white noise as a seed, generate a lum-PR image which has the luminance amplitude spectrum equal to that of the target. (2) Decompose both the target and the lum-PR image into orientation and spatial-frequency subbands through bandpass filters. (3) Convert Each subband into an energy image. (4) Perform four-dimensional fast-Fourier transform (4D-FFT) on the energy data to obtain the amplitude spectrum of the target and the phase spectrum of the lum-PR image. (5) Apply an inverse FFT to the amplitude and phase spectra to obtain new subband energy data. (6) Extract linear subbands from energy data, and then collapse subbands to reconstruct the new luminance image.

It is well known that the luminance histogram, or pixel moment statistics, also has an impact on the appearance of a texture (Chubb et al., 1994; Balas, 2006; Motoyoshi et al., 2007). Most texture synthesis algorithms make use of this to get better results, and we found it to be true for the lum-energy PR images. Therefore, (7) we finally matched the luminance histogram of the lum-energy PR image to that of the target image. (8) The algorithm was iterated by replacing the initial seed with the obtained image to modify the distortion of the spectral shape caused by the histogram matching; after about 20 iterations, the perceptual changes converged in most cases.

**FIGURE 5:**
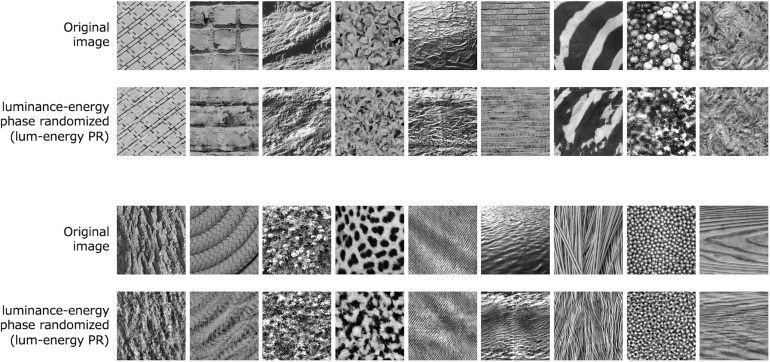
Luminance-energy phase randomized images of various natural textures.

We applied the lum-energy phase randomization for 300 natural textures. Figure 5 shows examples of the results, indicating that the lum-energy PR images duplicate the characteristic appearance of each natural texture, even though they only share the 1st- and 2nd-order spectra. We have also confirmed that this randomization works well for several types of artificial textures that require higher-order (3rd- or 4th-order) statistics to be discriminated (e.g., Chubb et al., 1994; Victor et al., 2005). We also observed that the lum-energy PR, while particularly effective for strongly periodic textures such as tiles and bricks, seems to fail for textures with complex shading patterns such as bumpy surfaces under directional lighting.

**FIGURE 6:**
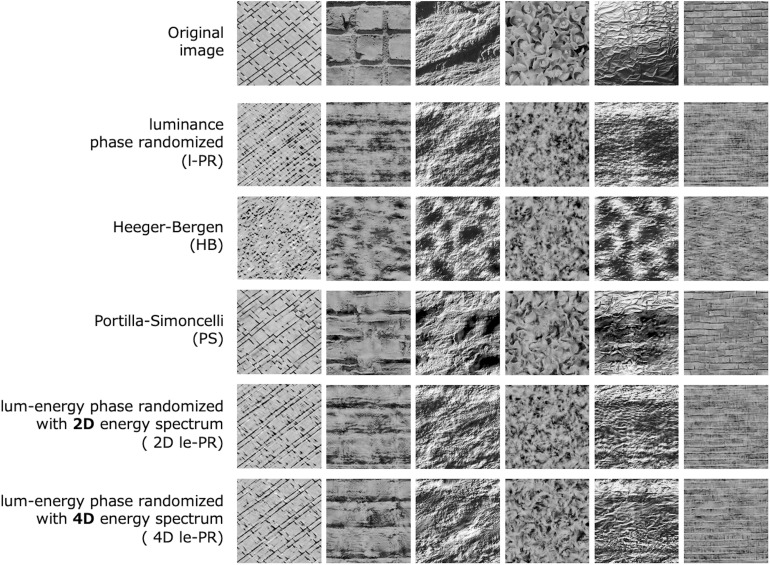
Comparison of the images of lum-energy PR (4D le-PR) with the images of luminance phase randomization (l-PR), Heeger-Bergen synthesis (HB), Portilla-Simoncelli synthesis (PS), and 2D lum-energy PR (2D le-PR).

Figure 6 compares the lum-energy PR images (4D le-PR) with images synthesized by other methods. For this we chose four algorithms: the classical luminance phase randomization with luminance histogram matching (l-PR), the Heeger-Bergen (HB) texture synthesis (Heeger and Bergen, 1995), the Portilla-Simoncelli texture synthesis (PS), and 2D lum-energy PR (2D le-PR). Here, 2D le-PR is a phase randomization with the energy spectrum obtained by the 2D-FFT only across space, instead of the 4D-FFT across space, orientation, and spatial frequency. In other words, 2D le-PR is based on a model corresponding to the conventional FRF not considering the correlation between orientation and spatial frequency. The synthesis algorithm of 2D le-PR is exactly the same as that of 4D le-PR except for the FFT dimension. We added 2D le-PR to see the difference in the synthesized textures when the 2nd-order spectral analysis is done in 2D as in the FRF model and when it is done in 4D as according to our idea. At least for the samples shown in Figure 6, the 2D and 4D le-PR images appear similar at first glance. However, a closer look reveals that 4D le-PR captures the detailed features a little better, and our psychophysical experiment with 300 textures described below showed that 4D le-PR was significantly better than 2D le-PR in terms of the perceptual similarity to the original image.

**FIGURE 7:**
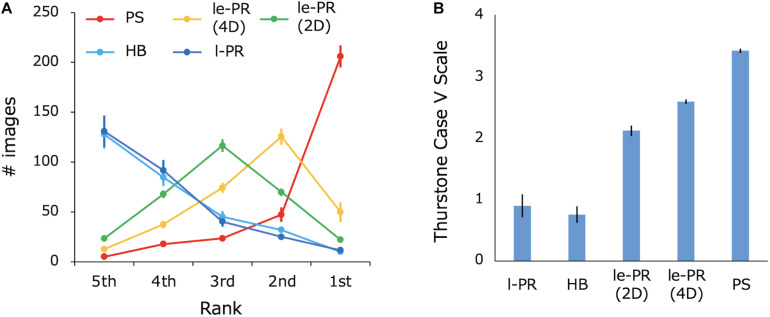
**(A)** The number of images selected for each rank. **(B)** The relative similarity to the original texture as scaled by Thurstone’s method. The value is additively normalized with a minimum of 0. Error bars represent ±1 SEM across eight observers.

To compare the perceptual quality of the lum-energy PR textures with those of the other synthetic textures, we had human observers assess the perceptual similarity to the original for natural textures of 300 samples, which is much larger than the number of samples used in previous studies (Balas, 2006; Wallis et al., 2017). Eight observers ranked the perceptual similarity of five synthetic images obtained with different methods and the original natural texture. Figure 7A shows the average number of images selected for each rank. 4D le-PR was most frequently ranked second. PS synthesis was most frequently ranked first and had the best overall results. 2D le-PR was most frequently ranked third, and 4D le-PR was often ranked higher than 2D le-PR. L-PR (with histogram matching) and HB synthesis had about the same number of images in all ranks and were less likely to be ranked highly. The number of times that 4D le-PR ranked first over PS was significantly higher than the numbers of times that the other three methods ranked first [one-sided Welch’s *t*-test, *t*(13) > 3.01, *p* < 0.006, *d* > 1.51], indicating a high degree of perceptual similarity for 4D le-PR. Figure 7B shows the relative perceptual similarity to the original, which was scaled using Thurstone’s method Case V (Thurstone, 1927): for each synthesis method, the scale values are calculated as the sum of the log-transformed winning percentages against the other syntheses. The results show that, on average, the 4D le-PR image is inferior to the PS image but better than the images of 2D le-PR, l-PR, and HB. In particular, there was a statistically significant difference between 4D le-PR and 2D le-PR [one-sided Welch’s *t*-test, *t*(10) = 4.68, *p* < 0.001, *d* = 2.34].

Although we did not control stimulus duration, if we controlled it to a short time, the importance of the statistics (and hence the rank between synthesis conditions), might have changed due to temporal dynamics in the hierarchy of neural processing.

## Discussion

In the present study, we extended the dimensions of FRF processing and proposed a novel model that texture perception is based on the 1st-order (2D-luminance) and 2nd-order (4D-energy) amplitude spectra of the image. The model is represented within only two single spectral spaces (+pixel histogram), and it provides a simple framework to describe and predict texture representations in various visual tasks, including scene and material perception. In addition, the notion is consistent with the PS statistical model, and it therefore provides a comprehensive understanding of the FRF and PS models in the frequency domain.

The model is biologically plausible as both the FRF and PS models are supported by rich physiological correlates in the early visual cortex, such as simple and complex cells in V1 (Hubel and Wiesel, 1968), spatial and sub-spatial neural interactions in V1 (Morrone, et al., 1982; Ohzawa et al., 1982; Zipser et al., 1996; Ringach et al., 1997; Nishimoto et al., 2006), second-order neurons in V2 (Baker and Mareschal, 2001), and image statistics coding in V1 and V2 (Freeman and Simoncelli, 2011; Ziemba et al., 2016). Spatial pooling of these signals within a large receptive field, which represents global image statistics in the PS model and global energy spectrum in the present model, are likely to be implemented in V4 neurons (Okazawa et al., 2015). As for the analysis of the 2nd-order spectrum, one can assume that the neuronal unit (probably in V2) with 4D receptive fields analyzes the inputs (probably from V1) over space, orientation, and spatial frequency. Notably, such a neural circuit is physiologically sensible given the functional architecture of V1 in which neurons tuned to the spatial position, orientation, and spatial frequency are regularly mapped along the cortical surface (Hubel and Wiesel, 1968; Grinvald et al., 1986; Nauhaus et al., 2012). However, given the fact that such neural interactions are generally local, it is unlikely that the global spectrum, decomposed into localized frequencies as assumed in the present notion, is represented neuronally. In this respect, the idea of a two-stage spectrum provides a simple understanding but remains problematic in terms of physiological plausibility.

The model analyzes up to the 2nd-order spectrum: the final output is a pooled summary of the 2nd-stage (i.e., global spectrum analysis), and no further analysis is performed. Termination of the process at the 2nd-stage is based on the notion that relatively low-level features are important for preattentive texture perception. However, it is also possible to perform a local spectral analysis without pooling in the 2nd-stage, as in the 1st-stage, and continue the spectral analysis at higher stages. Such an extension may reconcile the findings that point to the significance of higher-order features in texture perception (Julesz, 1981; Motoyoshi and Kingdom, 2010), although we did not directly examine this.

One may notice that such a multi-order spectral analysis is remarkably consistent with the hierarchical processing principle of the visual brain (Van Essen and Maunsell, 1983). The recent success of deep neural networks (DNN) largely depends on multiple layers of convolution and non-linear pooling, which mimic neural computation in the visual cortex (Fukushima and Miyake, 1982; Riesenhuber and Poggio, 2000; LeCun et al., 2015). In a typical DNN for visual object recognition, the 1st layer is characterized as a bank of filters that extract orientation and spatial frequency components from the image (LeCun et al., 2015). The 2nd layer is assumed to be a filtering of the rectified and pooled outputs from the 1st layer. If the network is limited to two layers, these computations are analogous with the two-stage spectral analysis. This in turn leads us to suggest that the standard form of a convolutional network can be generally formalized as a “multi-order local spectral analyzer” which continuously performs local spectral analysis on the data form the previous layer. Our idea of a two-stage spectrum explains texture perception through spectral analysis up to the 2nd-order, but if we continue the analysis beyond the 3rd-order, it may work as a generalized computational model for a wider range of visual functions, including object and scene recognition.

It should also be mentioned there are some discoveries that have a similar structure to our model. One of those examples is the wavelet scattering network used to compute a translation-invariant image representation for classification (Mallat, 2012; Bruna and Mallat, 2013). This framework consists of an iterative process of filtering and energy measurement on the output of the previous stage, which is a form common to the spectral analysis extended to higher-order stages. However, in the wavelet scattering network, the analysis is always applied to a two-dimensional output. In our model, by comparison, the number of dimensions to be analyzed increases as the stages go higher. In the wavelet scattering network, energy converges rapidly to zero as order increases, and for most applications, a network up to the second order is usually considered sufficient (Bruna and Mallat, 2013). It is an intriguing coincidence that the texture vision models can account for the human perception by using up to a 2nd-order process.

Furthermore, the analogy of the two-stage spectral analysis applies not only to vision but also to audition. One good example is the analysis of the modulation spectrum of natural sounds (Singh and Theunissen, 2003). That study’s analysis of the envelopes of natural sounds by a two-dimensional Fourier transform of time and frequency strongly ties into our idea of a four-dimensional spectrum of energy. Another model, the powerful natural-sound synthesis methods by McDermott and Simoncelli (2011) also incorporate calculations of subband envelope modulation and highlight the importance of frequency analysis of 1st-order output. Taken together, this leads to the possibility that multi-order spectrum analysis is a universal form of cortical computation of texture information across sensory modalities.

While we introduced the luminance-energy phase randomization (lum-energy PR) only to test the idea of the two-stage spectrum, it may be used as a new technique to synthesize naturalistic textures. The algorithm is simpler than PS synthesis as it is mainly based on the FFT and histogram matching only. On the other hand, the (4D) lum-energy PR requires a relatively large amount of data (total data = [N × N](histogram matching) + [N/2 × N/2](1st-order spectrum) + [N/2 × N/2 × 4 × 4](2nd-order spectrum), if N × N pixels of image size, eight orientations, and eight frequencies) because it was not designed to represent a texture image with a compact code. However, there is space to compress the data size by using under-sampling, PCA, ICA, etc. As the data are represented only in two single spaces (i.e., 2D spectrum and 4D spectrum), one would apply PCA/ICA more effectively than previously done for the PS statistics (Okazawa et al., 2015). With regard to the synthesis quality under free viewing, neither PS synthesis nor lum-energy synthesis outperform recent CNN-based methods (Gatys et al., 2015); note that PS synthesis matches or outperforms CNN-based textures when briefly presented in the near periphery (Wallis et al., 2017). Nevertheless, these methods would still be useful to understand specific neural computations involved in texture perception.

The psychophysical results show that there is a significant difference in the synthesis quality of the lum-energy PR texture depending on whether the preserved energy spectrum is obtained by 4D-FFT or 2D-FFT. The improvement in representation is considered one of the advantages of extending the conventional FRF model that operates only in the spatial dimension to our model that also considers orientation and spatial frequency correlations. It is noted, however, that the difference was small when compared with the difference between PS synthesis and 4D le-PR. This suggests that the effect of energy correlation across orientation and frequencies on the quality of the synthesis is not larger that of energy correlation across space.

Through the development of lum-energy PR images, we also found that the pixel-luminance histogram plays a significant role in addition to the two spectra data. This is consistent with the previous texture models, including PS (Portilla and Simoncelli, 2000; Balas, 2006) and HB (Heeger and Bergen, 1995).

### Methods: Luminance-Energy Phase Randomization

Luminance-energy phase-randomized images were generated according to the following procedure. All computations were implemented by a MATLAB code. An image with the same luminance amplitude spectrum as the target (lum-PR image) was generated using white noise as a seed. Both the target and the lum-PR image were decomposed into subband images with eight orientations (0–157.5° in 22.5° step) and eight spatial frequencies (1–128 cycle/image in 1 octave step) using log-Gabor filters with a spatial-frequency bandwidth of 1 octave and an orientation bandwidth of 30°. Each subband was then converted into an energy image by taking the square root of the sum of squares of the quadrature pair. The amplitude spectrum of the target energy and the phase spectrum of the lum-PR image energy were then obtained by four-dimensional fast-Fourier transform (4D-FFT) on the energy data. New subband energy data was obtained by the inverse FFT of the amplitude and phase spectra. Linear subbands were extracted from energy data using the carrier from the lum-PR image. A new luminance image was then obtained by collapsing the linear subbands. Finally, a luminance histogram of the obtained image was matched to that of the target. Histogram matching was performed in the same way as in the Heeger-Bergen synthesis (Heeger and Bergen, 1995). The whole algorithm was iterated 20 times, with the initial being replaced with the histogram-adjusted image on each iteration.

### Methods: Psychophysical Experiment

Visual stimuli consisted of 300 natural texture images (4.3 × 4.3 deg, 256 × 256 pixels). They were collected from NYU Laboratory for Computational Visiony^1^, McGill Calibrated Color Image Database^2^ (Olmos and Kingdom, 2004), and our own database. Heeger-Bergen synthesis and Portilla-Simoncelli synthesis were carried out using the original algorithm (Heeger and Bergen, 1995; Portilla and Simoncelli, 2000). The lum-energy PR (4D, default) was carried out using the algorithm described above. The 2D lum-energy PR was carried out by replacing 4D-FFT with 2D-FFT in the algorithm. This 2D-FFT was applied only across x-y space for each orientation and frequency subband. The lum PR image was generated by iterating alternately the phase randomization of luminance and histogram matching. Algorithms were iterated 20 times for all methods except Heeger-Bergen synthesis. For Heeger-Bergen synthesis only, the number of iterations was set at five as recommend by the original paper.

In each trial, the original texture was presented in the center of the background, and synthetic textures from the five different methods were randomly presented at each vertex of a regular pentagon with the original as the center, and all were located at 6.0° from the center. The observers viewed the display with free gaze and ranked the perceptual similarity of the synthetic images to the original image. Stimuli were shown until the observer responded.

One of the authors and seven naïve paid volunteers participated in the experiment (one females, 21–28 years old, mean = 23.0, SD = 2.35). All of them had normal or corrected-to-normal vision. All experiments were conducted in accordance with the Ethics Committee for Experiments on Humans of the Graduate School of Arts and Sciences, The University of Tokyo. All stimuli were generated by a PC and presented on LCD or OLED monitors with a refresh rate of 60 Hz. Due to the COVID-19 pandemic situation, each observer used LCD monitors (BenQ XL2720B, BenQ XL2730Z, BenQ XL2735B, and BenQ XL2430T) or OLED monitors (SONY PVM-A250 and SONY PVM 2541A) installed in a dark room at their individual homes. The luminance of all monitors was carefully calibrated and gamma-corrected by Colorimeter (ColorCal II CRS). The mean background luminance ranged from 26.2 to 48.6 cd/m^2^ (mean = 36.2, SD = 7.48). The viewing distance was adjusted so that the pixel resolution was 1.00 min/pixel. The size of the background in each monitor varied from 31.0° (W) × 18.0° (H) to 42.7° (W) × 24.0° (H).

## Data Availability Statement

The original contributions presented in the study are included in the article, further inquiries can be directed to the corresponding author.

## Ethics Statement

The studies involving human participants were reviewed and approved by the Ethics Committee for Experiments on Humans of the Graduate School of Arts and Sciences, The University of Tokyo. The patients/participants provided their written informed consent to participate in this study.

## Author Contributions

IM conceived the study. KO and IM designed the study and experiment and wrote the manuscript. KO collected and analyzed the data. Both authors contributed to the article and approved the submitted version.

## Conflict of Interest

The authors declare that the research was conducted in the absence of any commercial or financial relationships that could be construed as a potential conflict of interest.

## Publisher’s Note

All claims expressed in this article are solely those of the authors and do not necessarily represent those of their affiliated organizations, or those of the publisher, the editors and the reviewers. Any product that may be evaluated in this article, or claim that may be made by its manufacturer, is not guaranteed or endorsed by the publisher.

## Footnotes

1 http://www.cns.nyu.edu/~eero/software.html

2 http://tabby.vision.mcgill.ca/
